# Comparison of Latest Generation Intra‐ and Supra‐Annular Self‐Expanding Transcatheter Heart Valves for Aortic Valve‐in‐Valve Procedures

**DOI:** 10.1002/ccd.70011

**Published:** 2025-07-17

**Authors:** Antonia Katharina Stötzel, Oliver D. Bhadra, Lara Waldschmidt, Till J. Demal, David Grundmann, Lisa Voigtländer, Stefan Blankenberg, Hermann Reichenspurner, Niklas Schofer, Andreas Schaefer

**Affiliations:** ^1^ Departments of Cardiovascular Surgery University Heart and Vascular Center Hamburg, University Hospital Hamburg Eppendorf Hamburg Germany; ^2^ Departments of Cardiology University Heart and Vascular Center Hamburg, University Hospital Hamburg Eppendorf Hamburg Germany

**Keywords:** AVD—aortic valve disease, SDES—stent design/structure/coatings, TVI—transcatheter valve implantation

## Abstract

**Background:**

Valve‐in‐valve (ViV) procedures are an established therapy. Self‐expanding (SE) supra‐annular transcatheter heart valves (THV) achieve superior hemodynamics compared to balloon‐expandable (BE) intra‐annular THV, but influence of stent design versus valve position remains unclear.

**Aims:**

This study compares SE intra‐ and supra‐annular THV for aortic ViV procedures to further elucidate this topic.

**Methods:**

From 05/2022 to 11/2023, 10 patients underwent ViV procedures with the SE intra‐annular Navitor valve. A control group of 22 patients treated with the SE supra‐annular Evolut R/PRO valve during the same period was retrieved from our database. Data were retrospectively analyzed according to updated valve academic research consortium (VARC) 3 definitions. A subgroup analysis matched patients for true internal diameter (ID) and body mass index (BMI).

**Results:**

Postprocedural transvalvular gradients were similar between groups. No differences in rates of significant paravalvular leakage (PVL) were seen (*p* = 0.131). No all‐cause mortality occurred in either group. Device success (study group, Navitor: 70 [7] vs. control group, Evolut: 90.1 [20]; *p* = 0.293), early safety (90 [9] vs. 86.4 [19]; *p* = 1.000), permanent pacemaker implantation (PPI) (0 [0] vs. 9.1 [2]; *p* = 1.000), and stroke (0 [0] vs. 4.5 [1]; *p* = 1.000) were similar between groups. Procedure time was shorter for the study group (65.8 ± 27.9 min vs. 145.7 ± 51.3 min; *p* < 0.001). After matching for True ID and BMI these results persisted.

**Conclusion:**

Intra‐ and supra‐annular THV showed similar clinical and hemodynamic outcomes in aortic ViV procedures. These findings highlight the applicability of SE intra‐annular THV for aortic ViV procedures.

**Abbreviations**
AKIN: acute kidney injury network
BASILICA: bioprosthetic or native aortic scallop intentinal laceration to prevent iatrogenic coronary artery obstruction
BE: balloon‐expanding
BMI: body‐mass‐index
BVF: bioprosthetic valve fracture
EOA: effective orifice area
ID: internal diameter
LA: local anesthesia
MSCT: multi‐slice computed tomography
PPI: permanent pacemaker implantation
PPM: patient‐prosthesis mismatch
PVL: paravalvular leakage
SE: self‐expanding
STS PROM: society of thoracic surgeons Predicted risk of mortality
TAVI: transcatheter aortic valve implantation
TEE: transesophageal echocardiography
TF: transfemoral
THV: transcatheter heart valve
TTE: transthoracic echocardiography
VARC: valve academic research consortium
ViV: valve‐in‐valve

## 1 Introduction

Transcatheter aortic valve implantation (TAVI) has become a cornerstone in the treatment of symptomatic severe aortic valve stenosis [1]. Moreover, TAVI in terms of Valve‐in‐valve (ViV) procedures is increasingly being utilized for the treatment of failing bioprosthesis and transcatheter heart valves (THV) [2, 3, 4, 5, 6, 7]. Especially when it comes to lifetime management of patients with aortic valve disease, ViV procedures are a curcial part of the therapeutic armamentarium. However, identification of periprocedural advantageous factors to achieve optimal long‐term results is of paramount importance for effective patient management [8].

One of the most important considerations in aortic ViV procedures comprises of postoperative hemodynamics and avoidance of patient‐prostheses mismatch (PPM) especially in patients with small bioprostheses and thus a small true internal diameter (True ID) [9, 10, 11]. Commonly, self‐expandable (SE) supra‐annular THV are considered to present superior hemodynamics after ViV compared to balloon‐expandable (BE) intra‐annular THV [12, 13, 14]. Correspondingly, the VIVID registry demonstrated a higher number of reinterventions in patients undergoing aortic ViV procedures using BE valves [15].

Yet, it remains uncertain whether this advantage is due to supra‐annular valve seating of common SE platforms or rather is a consequence of stent design properties [16, 17, 18, 19, 20].

In a first series we demonstrated that a latest generation SE intra‐annular device presents reasonable hemodynamic results and acute outcomes in ViV procedures [16]. However, there is a lack of comparative studies with supra‐annular platforms. We herein aimed to compare SE intra‐ and supra‐annular THV for ViV procedures for failing aortic bioprostheses.

## 2 Materials and Methods

### 2.1 Patients and Endpoints

Between May 2022 and November 2023 a consecutive series of 10 patients received transfemoral (TF) ViV‐TAVI for failing aortic bioprostheses using the SE intra‐annular Navitor (Abbott, Chicago, IL, US) (75.7 ± 11.8 years, 30% female, Body‐Mass‐Index [BMI] 23.9 ± 4.8 kg/m², Euroscore II 8.9% ± 5.2%, Society of thoracic surgeons Predicted risk of mortality score [STS PROM] 4.9 ± 3.6%). For a comparative assessment 22 patients treated with the SE supra‐annular Evolut R/PRO (Medtronic, Minneapolis, Minnesota) (79.9 ± 8.1 years, 50% female, BMI 26.9 ± 5.5 kg/m², Euroscore II 14.2% ± 8.9%, STS PROM score 5.1% ± 3.4%) during the same time frame were retrieved from our dedicated hospital database. In all cases the allocation of patients to TAVI followed current international recommendations and the indication was discussed by the local multidisciplinary heart team. Consensus regarding TAVI approach was achieved in all cases. There were no specific anatomical criteria for selecting either valve. The final decision on prosthesis type and size, as well as whether to perform bioprosthetic valve fracture (BVF) or bioprosthetic or native aortic scallop intentinal laceration to prevent iatrogenic coronary artery obstruction (BASILICA) was left to operator's discretion. All patients provided written informed consent before the procedure. Clinical endpoints were adjudicated in accordance with the updated standardized valve academic research consortium (VARC) 3 definitions [21]. The primary outcome of this study focused on postoperative valve hemodynamics (peak and mean transvalvular gradient, paravalvular leakage [PVL]) as assessed by the transthoracic echocardiography (TTE) at the time of discharge. Secondary outcomes included endpoints as defined by VARC‐3, followed up to the time of discharge.

### 2.2 Diagnostic Work‐Up

The preprocedural diagnostic work‐up and procedures followed institutional standards and were previously described [7, 22]: routine preprocedural diagnostics included preoperative TTE, as well as a coronary angiogram to evaluate the cardiac functional status. Moreover the work‐up involved contrast‐enhanced, electrocardiogram‐gated multislice computed tomography (MSCT). The computer tomography datasets were subsequently analyzed using 3mensio Medical Imaging software (3mensio, Medical Imaging, Bilthoven, Netherlands) to confirm bioprosthetic valve size including True ID, aortic root anatomy and morphology focusing on the distance from the coronary arteries to the bioprostheses or THV, sinus width, calcification patterns, and preprocedural calculation of optimal C‐arm angulation. Valve size was determined by perimeter and area values of the virtual annular plane on MSCT and the manufacturer's sizing chart [23].

Procedures were performed under local anesthesia (LA), conscious sedation (CS) or general anesthesia in a specially equipped hybrid operating room by a dedicated team of cardiac surgeons, cardiologists and anesthesiologists. THV function was assessed through invasive hemodynamic measurements, aortic root angiography and echocardiography [24].

### 2.3 Study Procedure

The institutional standards for aortic ViV procedures were previously described [25]. Briefly summarized, all herein described procedures were performed via TF access, LA was the first‐line approach. In patients requiering BASILICA, general anesthesia was used to enable transesophageal echocardiography (TEE) guidance.

Utilized vascular closure systems consisted of suture‐based devices (ProGlide/ProStyle; Abbott, Chicago, IL, USA) or a collagen plug‐based device (MANTA; Teleflex, Wayne, PA, USA). In all patients without BASILICA, a single femoral puncture was performed for interventional access, and non‐interventional access was established via the right radial artery. A cerebral embolic protection system (Sentinel, Boston Scientific, Marlborough, MS, US) was placed in two cases via the right radial artery into the brachiocephalic trunk and the left common carotid artery to protect against potential distal embolization.

The target height for THV valve deployment was the alignment of both lower stent rims to ensure optimal postinterventional hemodynamics with full deployment of the THV stent. BASILICA and BVF were performed as previously described [26, 27].

Following the procedure, all patients were transferred to a postoperative holding area until the first postoperative day and subsequently stayed in the ward until discharge.

### 2.4 Statistical Analysis

Data aquisition was performed anonymized and retrospectively. Therefore in accordance with German law, no ethical approval is needed and informed patient consent was waived. Clinical data were prospectively gathered and retrospectively analyzed. Data is presented as percentages and absolute numbers for categorical variables and as mean values with standard deviation, or median and interquartile range for continuous variables, as specified. For comparison between groups regarding True ID of the index valve and BMI, the best possible matches were determined before analysis. Categorical data comparisons were performed using the Fisher's two‐tailed exact test. Continuous data comparisons between the two groups were conducted using either the unpaired *t*‐test or the Mann−Whitney *U* test, as appropriate, based on variable distribution. To ensure that the assumptions for the *t*‐test were met, data was partially (log) transformed following testing and generating histograms of each variable. A two‐sided *p* value of <0.05 was considered statistically significant for all analyses. All computations were carried using the statistical software IBM SPSS 29.0.2: SPSS‐29‐64‐Bit (with integrated FixPack).

## 3 Results

### 3.1 Baseline Demographics

The final overall cohort compromised 32 patients (SE intra‐annular, group 1: 10 patients; SE supra‐annular, group 2: 22 patients). Both groups did not differ regarding baseline characteristics (age: group 1: 75.7 ± 11.8 years vs. group 2: 79.9 ± 8.1 years; *p* = 0.253; gender distribution: 30% female vs. 50% female; *p* = 0.446). Also, values of common risk stratification models presented no differences between groups (Euroscore II [8.9 ± 5.2% vs. 14.2 ± 8.9%; *p* = 0.061;] STS PROM score 4.9 ± 3.6% vs. 5.1 ± 3.4%; *p* = 0.938). Structural valve deterioration was identified as the mode of valve failure in all cases, except for one patient in the study group who presented a PPM as a non‐structural valve dysfunction.

A comprehensive summary of baseline demographics is provided in Table 1.

**Table 1:** Baseline data.

**Baseline data**		**Study group**	**Control group**	∆	*p* value
		*N* = 10	*N* = 22		
Age, years		75.7 ± 11.8	79.9 ± 8.1	−4.2	0.253
Female gender		30 (3)	50 (11)	−20	0.446
BMI, kg/m²		23.9 ± 4.8	26.9 ± 5.5	−3	0.141
Euroscore II,%		8.9 ± 5.2	14.2 ± 8.9	−5.3	0.061
STS PROM score, %		4.9 ± 3.6	5.1 ± 3.4	0.2	0.938
Index bioprosthesis size, mm		25 [23; 26]	23 [21; 25]	1.2	0.251
Time to ViV, months		142.2 ± 57.2	156.4 ± 18.2	−14.2	0.577
Mode of valve failure^a^	1	90 (9)	100 (22)	−10	0.138
	2	10 (1)	0 (0)	0	
Arterial hypertension		80 (8)	81.8 (18)	−1.8	1.0
Prior stroke		0 (0)	4.5 (1)	−4.5	1.0
Coronary artery disease		80 (8)	81.8 (18)	−1.8	1.0
Extracardiac atheropathy		10 (1)	27.3 (6)	−17.3	0.387
Arrhythmia		50 (5)	63.6 (14)	−13.6	0.699
COPD^b^ > Gold II		10 (1)	9.1 (2)	0.9	1.0
Creatinine, mg/dl		1.2 ± 0.6	1.2 ± 0.3	0	0.593
Pulmonary hypertension > 60 mmHg		0 (0)	4.5 (1)	−4.5	1.0
NYHA ≥ III		60 (6)	45.5 (10)	14.5	0.704
LVEF, %		52.5 ± 14.6	51.6 ± 11.1	0.9	0.471

*Note:* Values are % (*n*), mean ± SD or median [IQR]. The gray shades are solely for clarity and structuring purposes; the two different colour tones help visually distinguish between the two cohorts.

Abbreviations: BMI, body mass index; COPD, chronic obstructive pulmonary disease; Euroscore, European System for Cardiac Operative Risk Evaluation; LVEF, left ventricular ejection fraction; NYHA, New York Heart Association; PROM, predicted risk of mortality; STS, Society of Thoracic Surgeons; ViV, valve‐in valve.

^a^ according to VARC‐3 definitions (1: Structural valve deterioration, 2: Non structural valve dysfunction [patient‐posthesis‐mismatch, paravalvular regurgitation, other], 3: thrombosis, 4: endocarditis).

^b^ COPD according to EuroSCORE definitions.

### 3.2 Periprocedural Data

Baseline peak and mean gradients showed no differences between study and control group (53.8 ± 28.7 mmHg vs. 57.9 ± 25.6 mmHg; *p* = 0.696/32.8 ± 17.1 mmHg vs. 33.7 ± 17.2 mmHg; *p* = 0.889). There was a tendency toward higher invasive pre‐implant peak gradients in the SE intra‐annular cohort (52.8 ± 13.2 mmHg vs. 42.2 ± 27.2 mmHg; *p* = 0.324) without reaching statistical significance. However, the invasive pre‐implant mean gradient was higher in the study group (56.8 ± 5.7 mmHg vs. 40.6 ± 17.2 mmHg; *p* = 0.017). Rate of aortic regurgitation ≥ 2 was similar between groups (50 [5] vs. 63.6 [19], *p* = 0.364). Majority of procedures were performed under LA (80 [8] vs. 50 [11]; *p* = 0.378), aligning with the higher incidence of BVF and BASILICA in the SE supra‐annular cohort. No significant differences regarding fluoroscopy time (17.1 ± 8.6 min vs. 27.7 ± 18.6 min; *p* = 0.064) or contrast agent usage (40.5 [25; 71] ml vs. 75 [48; 192] ml; *p* = 0.127) were seen. Procedure time was significantly shorter in the study group (65.8 ± 27.9 min vs. 145.7 ± 51.3; *p* < 0.001). True ID of the index valve (measured by MSCT) (21.5 ± 2.1 mm vs. 20.3 ± 2.4 mm; *p* = 0.191) and THV size (25 [23; 25] mm vs. 26 [23; 26] mm; *p* = 0.558) were similar between the groups. Re‐sheating (0 [0; 1], *n* [40%] vs. 1 [0; 1], *n* [60%]; *p* = 0.404), predilatation (20 [2] vs. 9.1 [2]; *p* = 0.572) and postdilatation (60 [6] vs. 68.2 [15]; *p* = 0.703) did not yield any different results. A cerebral embolic protection system was employed in two cases (10 [1] vs. 4.5 [1]; *p* = 0.534). BVF was performed on four patients (10 [1] vs. 13.6 [3]; *p* = 1.000), while BASILICA was conducted in six patients (0 [0] vs. 27.3 [6]; *p* = 0.142), with one patient undergoing both procedures concomitantly. The invasive post‐implant peak (5 [5; 9] mmHg vs. 7.5 [3; 13] mmHg; *p* = 0.754) and mean (13.2 ± 4.7 mmHg vs. 12.7 ± 5.9 mmHg; *p* = 0.869) gradients did not show any differences.

Detailed periprocedural data can be found in Table 2.

**Table 2:** Periprocedural data.

**Periprocedural data**		**Study group**	**Control group**	∆	*p* value
		*N* = 10	*N* = 22		
Baseline peak gradient, mmHg		53.8 ± 28.7	57.9 ± 25.6	−4.1	0.696
Baseline mean gradient, mmHg		32.8 ± 17.1	33.7 ± 17.2	−0.9	0.889
Invasive pre‐implant peak gradient, mmHg		52.8 ± 13.2	42.2 ± 27.2	10.6	0.324
Invasive pre‐implant mean gradient mmHg		56.8 ± 5.7	40.6 ± 17.2	16.2	**0.017**
Aortic regurgitation, grade	0	20 (2)	18.2 (4)	1.8	0.423
	1	30 (3)	18.2 (4)	11.8	
	2	30 (3)	27.3 (6)	2.7	
	3	20 (2)	36.4 (8)	−16.4	
Aortic regurgitation ≥ 2		50 (5)	63.6 (19)	−13.6	0.364
Anesthesia	LA	80 (8)	50 (11)	30	0.378
	CS	10 (1)	18.2 (4)	−8.2	
	general	10 (1)	31.8 (7)	−21.8	
Procedure time, min		65.8 ± 27.9	145.7 ± 51.3	−79.9	**<0.001**
Fluoroscopy time, min		17.1 ± 8.6	27.7 ± 18.6	−10.6	0.064
Contrast agent, ml		40.5 [25; 71]	75 [48; 192]	−30.8	0.127
True ID index valve (MSCT), mm		21.5 ± 2.1	20.3 ± 2.4	1.2	0.191
THV size, mm		25 [23; 25]	26 [23; 26]	−0.1	0.558
Re‐sheating, n		0 [0; 1]	1 [0; 1]	−0.2	0.404
Predilatation		20 (2)	9.1 (2)	10.9	0.572
Postdilatation		60 (6)	68.2 (15)	−8.2	0.703
Cerebral protection		10 (1)	4.5 (1)	5.5	0.534
Bioprosthetic valve fracture		10 (1)	13.6 (3)	−3.6	1.0
BASILICA		0 (0)	27.3 (6)	−27.3	0.142
Invasive post‐implant peak gradient, mmHg		7 ± 2.8	9.1 ± 7.6	−2.0	0.754
Invasive post‐implant mean gradient, mmHg		13.2 ± 4.7	12.7 ± 5.9	0.3	0.869

*Note:* Values are % (*n*), mean ± SD or median [IQR]. The bold numbers are these showing statistical relevant differences.

Abbreviations: BASILICA, Bioprosthetic or native aortic scallop intentinal laceration to prevent iatrogenic coronary artery obstruction; MSCT, contrast‐enhanced, electrocardiogram‐gated multislice computed tomography; THV, transcatheter heart valve; True ID, true internal diameter.

### 3.3 Echocardiographic and Clinical Outcome Data at 30 Days

**Figure 1:**
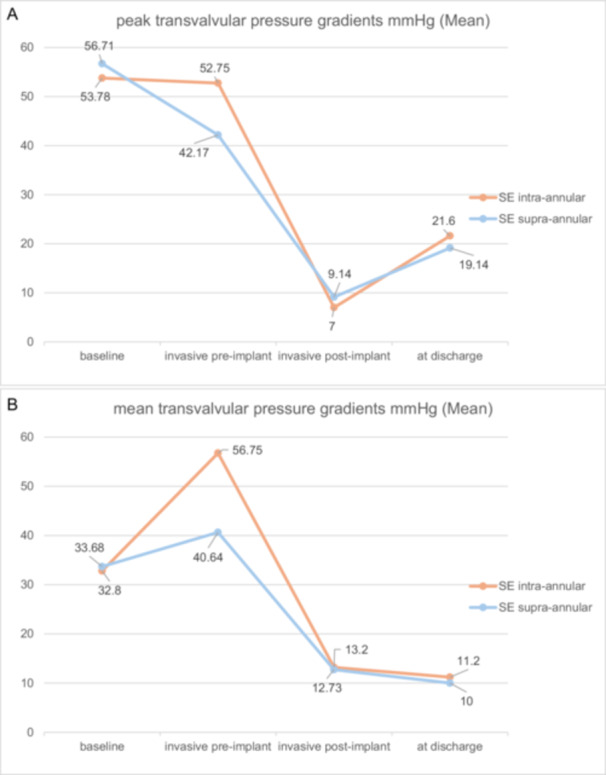
(A) Echocardiographic results of peak transvalvular pressure gradients in the course of treatment. (B) Echocardiographic results of mean transvalvular pressure gradients in the course of treatment. [Color figure can be viewed at wileyonlinelibrary.com]

**Figure 2:**
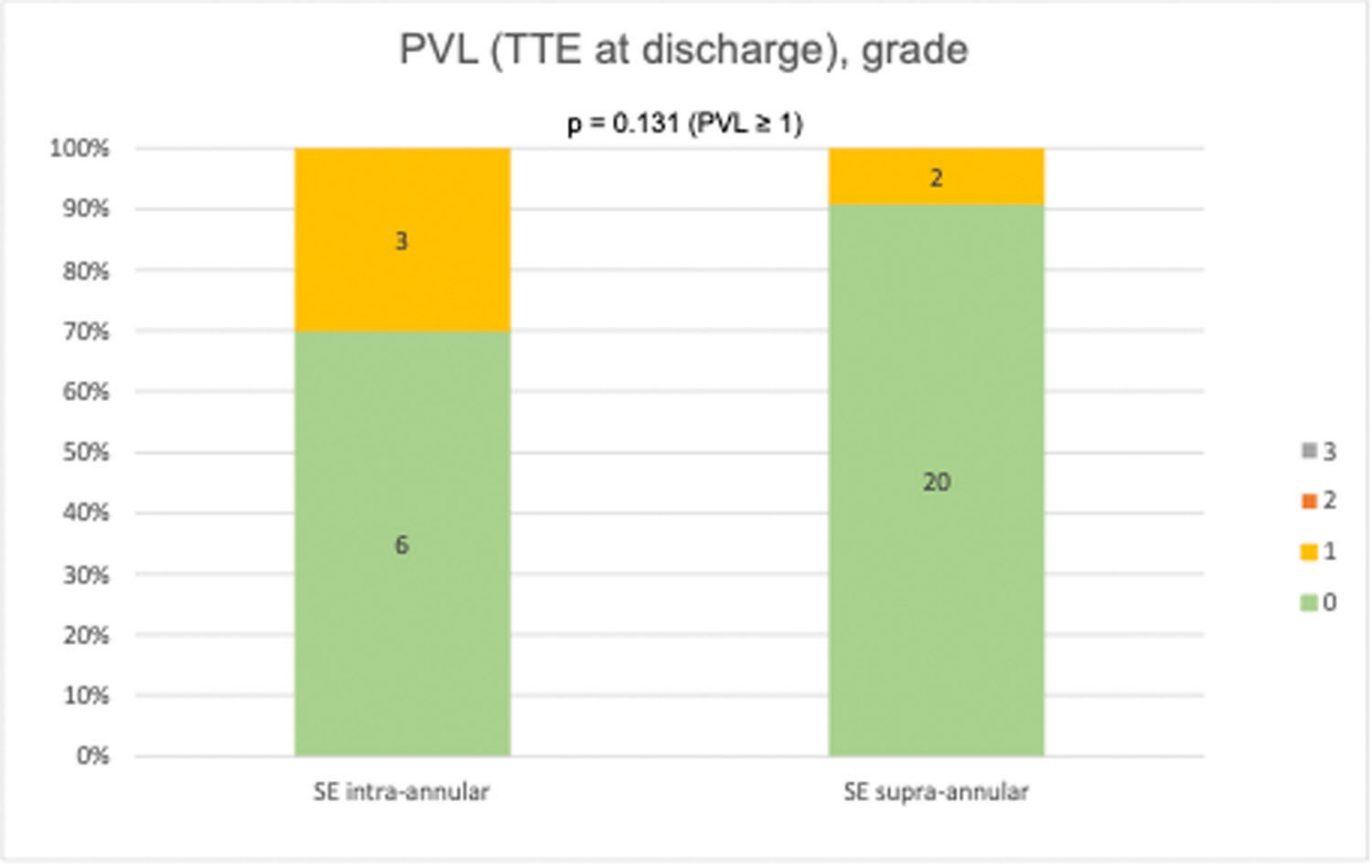
Distribution of PVL rates at discharge. PVL, paravalvular leakage; TTE, transthoracic echocardiography. [Color figure can be viewed at wileyonlinelibrary.com]

Device success was equal in both groups (70.0 [7] vs. 90.1 [20]; *p* = 0.293). Absence of device success occured due to a postprocedural transvalvular mean gradient ≥ 20 mmHg at time of discharge in two patients in the study group, and in two patients in the control group. In one patient in the study group implantation of a second THV was necessary. Due to this incident, the patient was excluded from the analysis when comparing postoperative hemodynamics. Early safety was similar between groups (90.0 [9] vs. 86.4 [19]; *p* = 1.000). Further reasons for absence of early safety were two permanent pacemaker implantations (PPI) (0.0 [0] vs. 9.1 [2]; *p* = 1.000) and one postprocedural stroke (0.0 [0] vs. 4.5 [1]; *p* = 1.000). None of the patients experienced all‐cause mortality, myocardial infarction, major or life‐threatening bleeding, major access site complications or acute kidney injury. Duration of stay in the holding area (1 ± 0 days vs. 1 ± 0 days) as well as the overall hospital stay (12.9 ± 5.3 days vs. 13.9 ± 6 days; *p* = 0.723) were similar between groups. The peak (21.6 ± 15 mmHg vs. 19.1 ± 11.5 mmHg; *p* = 0.621) and mean transvalvular gradient (11.2 ± 9.2 mmHg vs. 10 ± 6.5 mmHg; *p* = 0.736) measured by TTE at time of discharge demonstrated clinical efficacy of ViV procedures as shown in Figure 1. Additionally, there was no significant difference in trace/mild PVL as assessed by TTE at discharge (33.3 [3] vs. 9.1 [2]; *p* = 0.131), depicted in Figure 2. PVL > 1 did not occur in any patient.

Detailed clinical and echocardiographic results at discharge are displayed in Table 3.

**Table 3:** Clinical and echocardiographic results at discharge.

**Clinical and echocardiographic results at discharge**		**Study group**	**Control group**	∆	*p* value
		*N* = 10	*N* = 22		
Creatinine, mg/dL		1.1 ± 0.4	1 ± 0.2	0.1	0.984
All‐cause mortality (30 days)^d^		0 (0)	0 (0)	0	0
Cardiovascular or unknown^d^		0 (0)	0 (0)	0	0
Stroke^d^		0 (0)	4.5 (1)	−4.5	1.0
Myocardial infarction^d^		0 (0)	0 (0)	0	0
Bleeding (major/life threatening)^d^		0 (0)	0 (0)	0	0
Access site complications (major)^d^		0 (0)	0 (0)	0	0
Acute kidney injury (AKIN^a^ 2, 3)^d^		0 (0)	0 (0)	0	0
PPI^d^		0 (0)	9.1 (2)	−9.1	1.0
Device success^b^,^d^		70 (7)	90.1 (20)	−20.1	0.293
Early safety^c^,^d^		90 (9)	86.4 (19)	3.6	1.0
Holding area, days		1 ± 0	1 ± 0	0	0
In hospital stay, days		12.9 ± 5.3	13.9 ± 6	−1.0	0.723
Peak gradient (TTE at discharge), mmHg		21.6 ± 15	19.1 ± 11.5	2.5	0.621
Mean gradient (TTE at discharge), mmHg		11.2 ± 9.2	10 ± 6.5	1.7	0.736
PVL (TTE at discharge), grade	0	66.7(6)	90.9 (20)	−24.2	0.101
	1	33.3 (3)	9.1 (2)	24.2	
	2	0 (0)	0 (0)	0	
	3	0 (0)	0 (0)	0	
PVL (TTE at discharge) grade ≥ 1		33.3 (3)	9.1 (2)	24.2	0.131

*Note:* Values are % (*n*), mean ± SD or median [IQR].

Abbreviations: AKIN, acute kidney injury network; PPI, permament pacemaker implantation; PVL, paravalvular leakage TTE, transthoracic echocardiography.

^a^ AKIN Acute Kidney Injury Network.

^b^ Device success: technical success + absence of procedural mortality, correct positioning of a single prosthetic heart valve into the proper anatomical position, intended performance of the prosthetic heart valve (no prosthesis‐patient mismatch and mean aortic valve gradient < 20 mmHg or peak velocity < 3 m/s and no moderate or severe prosthetic valve regurgitation.

^c^ Early safety at 30 days: all‐cause mortality (at 30 days), all stroke (disabling and non‐disabling), life‐threatening bleeding, acute kidney injury stage 2 or 3 (including renal replacement therapy), coronary artery obstruction requiring intervention, major vascular complication, valve‐related dysfunction requiring repeat procedure (BAV, TAVI, or SAVR).

^d^ At the time of discharge.

### 3.4 Comparison of Outcomes After Matching Pairs for True ID of the Index Valve and BMI

Patients with THV as index valve and those who underwent BVF or BASILICA were excluded from this analysis.

After matching, BMI (study group, *n* = 7; 24.6 ± 5.1 kg/m² vs. control group, *n* = 7; 24.7 ± 3.0 kg/m²; *p* = 0.980), and True ID of the index valve (21.9 ± 2.3 mm vs. 21.6 ± 2.2 mm; *p* = 0.819) were similar between groups.

Procedure time (55.1 ± 25 min vs. 153.3 ± 39.7 min; *p* < 0.001) and the amount of contrast agent used (28.6 ± 22 mL vs. 109.3 ± 81.1 mL; *p* = 0.039) was lower in the study group. The SE intra‐annular cohort exhibited a trend toward higher invasive pre‐implant transvalvular peak (48.5 ± 10.6 mmHg vs. 41.8 ± 33.5 mmHg; *p* = 0.732) and invasive pre‐implant transvalvular mean (53 ± 0 mmHg vs. 35 ± 15.1 mmHg; *p* = 0.097) gradients.

**Figure 3:**
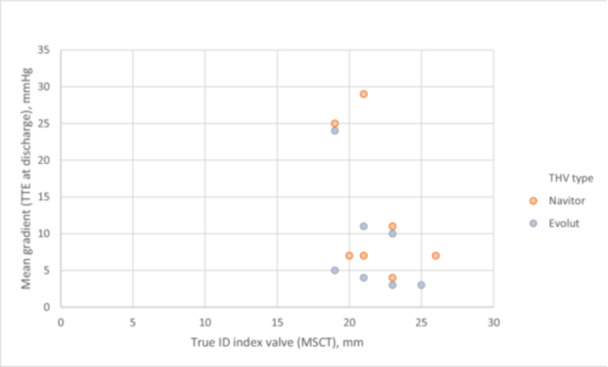
Comparison of the THV regarding mean transvalvular gradient measured via TTE at discharge after matching for True ID and BMI. BMI, body mass index; MSCT, contrast‐enhanced, electrocardiogram‐gated multislice computed tomography; THV, transcatheter heart valve; True ID, true internal diameter; TTE, Transthoracic echocardiography. [Color figure can be viewed at wileyonlinelibrary.com]

Secondary endpoints did not differ significantly in both cohorts. Device success was not achieved due to postprocedural mean gradients ≥ 20 mmHg, affecting three patients in total (71.4 [5] vs. 85.7 [6]; *p* = 1.000). Patients in the SE intra‐annular cohort presented similar peak (24.1 ± 16.2 mmHg vs. 16.4 ± 13.4; *p* = 0.244) and mean (12.9 ± 9.9 mmHg vs. 8.6 ± 7.5 mmHg; *p* = 0.381) gradient outcomes. Moreover, rate of PVL grade = 1 was comparable between both groups (42.9 [3] vs. 14.3 [1]; *p* = 0.280), along with complete absence of PVL > 1 in both groups. Illustration comparing the mean transvalvular gradient at time of discharge of matching pairs directly is shown in Figure 3. Detailed clinical and echocardiographic results at discharge are presented in Table 4.

**Table 4:** Clinical and echocardiographic results at discharge ‐ after matching for true internal diameter and body mass index.

**Clinical and echocardiographic results at discharge**		**Study group**	**Control group**	∆	*p* value
		*N* = 7	*N* = 7		
Creatinine, mg/dL		1.2 ± 0.4	1.1 ± 0.2	0.2	0.503
All‐cause mortality (30 days)^d^		0 (0)	0 (0)	0	0
Cardiovascular or unknown^d^		0 (0)	0 (0)	0	0
Stroke^d^		0 (0)	0 (0)	0	1.0
Myocardial infarction^d^		0 (0)	0 (0)	0	0
Bleeding (major/life threatening)^d^		0 (0)	0 (0)	0	0
Access site complications (major)^d^		0 (0)	0 (0)	0	0
Acute kidney injury (AKIN^a^ 2, 3)^d^		0 (0)	0 (0)	0	0
PPI^d^		0 (0)	0 (0)	0	0
Device success^b^,^d^		71.4 (5)	85.7 (6)	−14.3	1.0
Early safety^c^,^d^		100 (7)	100 (7)	0	0
Holding area, days		1 ± 0	1 ± 0	0	0
In hospital stay, days		12.4 ± 3.3	15.3 ± 6.7	−2.9	0.361
Peak gradient (TTE at discharge), mmHg		24.1 ± 16.2	16.4 ± 13.4	7.7	0.244
Mean gradient (TTE at discharge), mmHg		12.9 ± 9.9	8.6 ± 7.5	4.3	0.381
PVL (TTE at discharge), grade	0	57.1 (4)	85.7 (6)	−28.6	0.244
	1	42.9 (3)	14.3 (1)	28.6	
	2	0 (0)	0 (0)	0	
	3	0 (0)	0 (0)	0	
PVL (TTE at discharge) grade ≥ 1		42.9 (3)	14.3 (1)	28.6	0.28

*Note:* Values are % (*n*), mean ± SD or median [IQR].

Abbreviations: AKIN, acute kidney injury network; PPI, permament pacemaker implantation; PVL, paravalvular leakage; TTE, transthoracic echocardiography.

^a^ AKIN Acute Kidney Injury Network.

^b^ Device success: technical success + absence of procedural mortality, correct positioning of a single prosthetic heart valve into the proper anatomical position, intended performance of the prosthetic heart valve (no prosthesis‐patient mismatch and mean aortic valve gradient < 20 mmHg or peak velocity < 3 m/s and no moderate or severe prosthetic valve regurgitation.

^c^ Early safety at 30 days: all‐cause mortality (at 30 days), all stroke (disabling and non‐disabling), life‐threatening bleeding, acute kidney injury stage 2 or 3 (including renal replacement therapy), coronary artery obstruction requiring intervention, major vascular complication, valve‐related dysfunction requiring repeat procedure (BAV, TAVI, or SAVR).

^d^ At the time of discharge.

## 4 Discussion

This study represents the first direct comparison between SE intra‐ and supra‐annular THV for aortic ViV procedures. The main findings are as follows: (1) hemodynamic results were reasonable and similar in both groups, even in small bioprostheses without differences in postoperative transvalvular pressure gradients or rates of significant PVL. (2) Rates of secondary endpoints were low in both groups with complete absence of mortality, myocardial infarction and acute kidney injury. One patient in the SE supra‐annular group experienced postprocedural stroke and two patients required a PPI, slightly compromising early safety. (3) Procedures in the study group presented with significantly shorter procedure time, reduced contrast agent usage and shorter fluoroscopy time even after exclusion of BVF/BASILICA cases, suggesting potential ease of use for the SE intra‐annular THV. (4) After adjustment for commonly considered factors for PPM no significant differences were found between THV types for ViV procedures in terms of hemodynamic and clinical outcomes.

There is sufficient data supporting the advantages of SE supra‐annular devices over BE intra‐annular THV in ViV procedures. The ViViD Registry, encompassing 1006 patients, demonstrated significantly lower postprocedural peak and mean gradients for SE supra‐annular THV compared to BE intra‐annular THV. Higher gradients are associated with an increased risk of early valve deterioration and subsequent need for redo procedures, particularly when BE THV are used in smaller aortic bioprostheses, due to stent under‐expansion or valve thrombosis [15]. These findings emphasize the importance of lifetime management concepts when taking ViV procedures into consideration. Looking at the two most commonly used devices, BE intra‐annular THV such as the Sapien seem to be inferior compared to SE supra‐annular THV like the Evolut valve in terms of hemodynamic outcomes [9, 15, 28]. In contrast, the herein investigated SE intra‐annular Navitor device presented with similar hemodynamic results compared to the SE supra‐annular Evolut THV. Although, both the Navitor and Sapien THV are positioned intra‐annularly, our results suggest that differences in stent design and expansion mechanism may account for the performance deviations. Further advantages of using the investigated THV in ViV procedures may include improved coronary access due to the large stent cell design and facilitation of subsequent ViV‐in‐Valve procedures with supra‐annular valves by reduction of leaflet overhang and sinus sequestration [29, 30].

Secondary outcomes did not differ significantly between our patient cohorts with complete absence of mortality, myocardial infarction, major vascular complication, access site complications or AKIN, highlighting the clinical safety and efficacy of ViV procedures for deteriorated surgical and transcatheter bioprostheses in contemporary practice. Our results support findings of recent studys, suggesting high device success combined with low rates of PPI, PPM and mortality, especially for SE THV [31, 32, 33]. The previously observed high rates of PVL though could not be confirmed in our cohorts [34].

Moreover, when implementing the SE intra‐annular THV, the procedure time was significantly shorter with a reduction of contrast agent usage and fluoroscopy time. That implies an ease of use for the Navitor THV.

## 5 Limitations

Limitations for a retrospective single‐center study apply, including small sample size, absence of patient randomization and therefore possible bias by hidden confounders. However, the potential impact of learning curves however should be limited, as patients from both the study and control groups were treated during the same time period and no significant baseline differences were observed. Therefore, results should be interpreted with caution and outcomes should be evaluated in larger populations.

## 6 Conclusion

In this first series, intra‐annular SE THV present similar clinical and hemodynamic results in aortic ViV procedures compared to supra‐annular SE THV, even after matching for True ID and BMI. These results suggest a substantial impact of the stent design rather than valve seating on postprocedural hemodynamics after aortic ViV procedures. These findings support the applicabality of SE intra‐annular THVs for ViV procedures, potentially combining favorable hemodynamics with facilitation of a second ViV procedure. As our study includes a limited number of patients, these findings warrant further investigation in the future with a larger patient cohort.

## Ethics Statement

Data aquisition was performed anonymized and retrospectively. Therefore in accordance with German law, no ethical approval is needed and informed patient consent was waived.

## Conflicts of Interest

A.S. is a proctor for Abbott, received speaker honoraria and travel compensation from Edwards Lifesciences, Boston Scientific and Abbott. The other authors declare no conflicts of interest.

## Data Availability

The raw data is available from the corresponding author upon reasonable request.
